# Comparison of freeze-thaw and sonication cycle-based methods for extracting AMR-associated metabolites from *Staphylococcus aureus*

**DOI:** 10.3389/fmicb.2023.1152162

**Published:** 2023-04-27

**Authors:** Rita Singh, Lovnish Thakur, Ashok Kumar, Sevaram Singh, Shailesh Kumar, Manoj Kumar, Yashwant Kumar, Niraj Kumar

**Affiliations:** ^1^Translational Health Science and Technology Institute, NCR Biotech Science Cluster, Faridabad, India; ^2^Jawaharlal Nehru University, Delhi, India

**Keywords:** antimicrobial resistance, *Staphylococcus aureus*, Gram-positive, metabolites extraction method, freeze-thaw cycle, sonication cycle

## Abstract

Emerging antimicrobial resistance (AMR) among Gram-positive pathogens, specifically in *Staphylococcus aureus* (*S. aureus*), is becoming a leading public health concern demanding effective therapeutics. Metabolite modulation can improve the efficacy of existing antibiotics and facilitate the development of effective therapeutics. However, it remained unexplored for drug-resistant *S. aureus* (gentamicin and methicillin-resistant), primarily due to the dearth of optimal metabolite extraction protocols including a protocol for AMR-associated metabolites. Therefore, in this investigation, we have compared the performance of the two most widely used methods, i.e., freeze-thaw cycle (FTC) and sonication cycle (SC), alone and in combination (FTC + SC), and identified the optimal method for this purpose. A total of 116, 119, and 99 metabolites were identified using the FTC, SC, and FTC + SC methods, respectively, leading to the identification of 163 metabolites cumulatively. Out of 163, 69 metabolites were found to be associated with AMR in published literature consisting of the highest number of metabolites identified by FTC (57) followed by SC (54) and FTC + SC (40). Thus, the performances of FTC and SC methods were comparable with no additional benefits of combining both. Moreover, each method showed biasness toward specific metabolite(s) or class of metabolites, suggesting that the choice of metabolite extraction method shall be decided based on the metabolites of interest in the investigation.

## 1. Introduction

Antimicrobial resistance (AMR) among bacterial pathogens has become a leading cause of morbidity and mortality and hence a global public health concern demanding immediate action to develop strategies to combat such antimicrobial resistant difficult-to-treat bacterial infections (Dhingra et al., 2020). This recent trend indicates the emerging prevalence of Multidrug-resistant (MDR) is not only among Gram-negative (i.e., *Klebsiella pneumoniae*, *Acinetobacter baumannii*, and *Pseudomonas aeruginosa*, etc.) but also Gram-positive bacterial pathogens [primarily methicillin and gentamicin-resistant *Staphylococcus aureus* (MRSA)] making them also difficult to treat (Bassetti et al., 2019; Mulani et al., 2019). Tremendous efforts are being made to understand the pathogen and disease biology and develop relevant diagnostics and therapeutics for the effective treatment of Gram-negative pathogens; however, the same has been comparatively limited for Gram-positive pathogens (Vazquez-Guillamet and Kollef, 2014; Breijyeh et al., 2020).

World Health Organization (WHO) and the centers for disease control and prevention (CDC) have placed MRSA under a list of serious threat causing drug-resistant pathogens (WHO, 2021). MRSA alone has been responsible for more than 100,000 deaths worldwide in 2019 globally (Oxford, U.o, 2019). In India, the prevalence of MRSA has been recorded to be around 30–70% with high mortality rates among patients developing MRSA bacteremia (Antimicrobial Resistance, C, 2022). Originally, MRSA was common in the healthcare setting contributing to nosocomial/hospital-acquired (HA-MRSA) infections like those associated with surgical procedures, indwelling catheters, or prosthetic devices (Mehta et al., 2020). However, over the last decade, there has been an upsurge of community-associated MRSA (CA-MRSA) infections also like bone, joint and skin infections (Masimen et al., 2022). These HA- and CA-*S. aureus* infections are spread through direct contact with an infected wound or contaminated hands and can be fatal if remains untreated (Masimen et al., 2022). Further, limited therapeutic options and an increasing rate of emergence of AMR even against the available last-resort antibiotics have worsened the problem. Altogether, *S*. *aureus* infection is associated with a greater occurrence of complications, longer hospital stays, duration of therapy, as well as higher costs of treatment (Lee et al., 2018).

Therefore, understanding the molecular changes driving antibiotic resistance among Gram-positive bacteria, specifically *S*. *aureus*, has become critically important. This can be achieved using various new-generation biological tools, such as genomic, transcriptomic, proteomic, and metabolomics. However, metabolomics allows the identification and quantification of metabolites that are the end product of any genomic- and proteomic-based biological activity of an organism at a given point of time and hence provide a characteristic chemical fingerprint of a specific cellular process (Xiao et al., 2012). Recently, studies have shown that modulation of the pathogen’s metabolome can be used to deal with the emerging problem of AMR. One recent example is the conjugating antibiotics with small metabolites like aminoglycosides with fructose and fumarate, resulting in increased potency of aminoglycosides against *S. aureus* and *Escherichia coli* (Rosenberg et al., 2020). A central metabolite of the energy-generation pathway, pyruvate, has been reported to be associated with the virulence and pathogenicity of *S. aureus*, indicating it as a potential target for controlling infection (Harper et al., 2018). Further exogenous administration of L-valine, L-leucine, L-isoleucine, and L-proline in *S. aureus* bloodstream infection animal models has also been shown to have anti-infective effects (Pang et al., 2020). A decrease in intracellular ATP levels has been linked to the development of the *S. aureus* persister phenotype making them resistant to antibiotics (Conlon et al., 2016). However, existing knowledge of target(s) for regulating metabolomic modulation to treat resistant infections is still limited, owing to limited attempts to investigate the comprehensive metabolome of *S. aureus* (Kumar et al., 2022). This may potentially be due to the unavailability of the appropriate protocols and pipeline for investigating the metabolome of the Gram-positive bacteria, *S. aureus* (specifically drug-resistant strains), including the very first step of extracting AMR-associated metabolites.

Therefore, the goal of this study was to determine the optimal method for extraction of AMR-associated metabolites from *S*. *aureus* because the method of metabolite extraction dictates the possible range of metabolites that can be detected in the sample and thus has a large impact on the potential outcome(s) of the metabolomic studies. Most of the metabolomic investigations employ FTC or SC-based methods for extracting bacterial metabolites; however, their efficacy for extraction of AMR-associated metabolite from Gram-positive bacteria has not been investigated yet (Conlon et al., 2016). In this study, we have employed both methods, i.e., FTC and SC individually as well as their combination FTC followed by SC (FTC + SC) to extract the metabolites from the *S*. *aureus* pathogen. Further, Electrospray Ionization-Liquid Chromatography-Mass Spectrometry/Mass Spectrometry (ESI-LC–MS/MS; Orbitrap Fusion Tribrid, Thermo-Scientific), a highly sensitive and advanced mass-spectrometer was used to potentially increase the metabolomic coverage. Finally, the list of identified metabolites was subjected to an intensive literature search for finding the potential association of identified metabolites with AMR. Altogether, this information was utilized to identify the optimal metabolite extraction method for investigating AMR-associated metabolites.

## 2. Materials and methods

### 2.1. Harvesting of *Staphylococcus aureus* cell pellets

The primary culture of *S. aureus* (ATCC®33592) was inoculated in 5 ml Mueller Hinton Broth (MHB), followed by incubation for approximately 16 h at 37°C at 220 rpm. The secondary culture was inoculated (1% from primary culture) in fresh MHB and incubated till the exponential growth phase (Optical density, OD_600nm_:~0.6–0.7, i.e., 4 h post-inoculation) was achieved. Approximately 10^7^ cells (OD-based measurement) were harvested using centrifugation and washing with LC–MS grade cold water and kept at −80°C. The cell pellets were generated for 6 independent biological replicates. Since the cellular architecture and composition of member pathogens among a species are highly expected to be similar, a single strain of *S. aureus* was used for the study.

### 2.2. Bacterial metabolism quenching and extraction

For metabolite extraction, the stored cell pellet was thawed for 10 min on ice, followed by resuspension in 500 μl of mass spectrometry (MS) grade chilled methanol (Sigma). To evaluate the metabolites extraction efficiency of three extraction methods, i.e., FTC, SC, FTC + SC, 13C-labeled L-valine (40 ng/ml), an internal standard, was added to each sample before metabolite extraction (Kumar et al., 2022). For the freeze-thaw cycle-based metabolite extraction method, the resuspended cells were subjected to repeated freeze-thaw cycles (10 min at −80°C followed by 10 min on ice) three times. For the sonication-based metabolite extraction method, the mixture was sonicated for 2 min at 35 A° (10-s on-and-off cycles). Similarly, for FTC + SC, the resuspended cell pellet was subjected to a freeze-thaw cycle three times and followed by a sonication cycle as described above. The sample mixture following FTC, SC, or FTC + SC methods was collected as supernatant after centrifugation at 12,500 RPM for 10 min (4°C), aliquoted and stored at −80°C till further analysis. The metabolites were extracted using each method (FTC, SC and FTC + SC) from 6 independent biological replicate samples.

### 2.3. Separation and quantitation of metabolites using ESI-LC–MS/MS

The standard workflow for metabolite separation and measurement using ESI-LC–MS/MS was as followed. An aliquot (100 μl) of the sample was vacuum dried and the pellet was resuspended in 3:17, methanol: water mixture (25ul), followed by vortexing for 15 min, and 10 min centrifugation at 11000 rpm at 4°C. Data acquisition was done on C18 Reversed Phase HPLC Columns (C18) and Hydrophilic interaction LC (HILIC) columns with positive and negative mode separately. The collected metabolites were separated using C18 (HSS T3) and HILIC (XBridge BEH Amide) columns on UPLC ultimate 3,000 maintained at 40°C and 35°C temperature, respectively. A gradient of mobile phase A consisting of water +0.1% formic acid and mobile phase B (methanol + 0.1% formic acid) was used as a mobile phase for the C18 column. For the HILIC column, 20 mM ammonium acetate in the water of pH 9.0 (mobile phase A) and 100% acetonitrile (mobile phase B) were used for separation. For separation in the reverse phase, a gradient of 1% mobile phase B to 99% mobile phase B over 14 min (flow rate of 0.3 ml/min) was set and for the HILIC column, 85% mobile phase B to 10% B over 16 min (flow rate of 0.35 ml/min) was used. 5ul sample was injected into the column and for data acquisition. The Orbitrap Fusion Tribrid Mass Spectrometer (Thermo-Scientific) equipped with heated electrospray ionization (HESI) source was used for processing the sample using the following settings: 4000 positive mode spray voltage, 35,000 V for negative mode, 60–900 m/z mass range, AGC (Automatic gain control) was targeted at 100,000 ions. For data acquisition, 120,000 resolutions in MS1 mode and 30,000 resolutions in data-dependent MS2 scan mode were used. For MS, 50 ms was used as the maximum injection time while for MS/MS, an AGC target of 20,000 ions and a maximum injection time of 60 ms was used.

### 2.4. Metabolites identification and data analysis

The untargeted workflow of Progenesis QI software for metabolomics from Water Corporation was used at default settings for acquiring the data and its analysis. The MetaScope plugin of Progenesis QI metabolites was used for matching the mass, fragmented ions pattern and retention time of identified compounds with a list of 950 metabolites from our in-house library followed by an online spectral library search to confirm the identified metabolites. Peak detected in ≥4 replicates (out of 6 independent biological replicates) with intensity ≥100 (confidence interval->95%) receives an identification by spectral match. Further, ±1 min and 5 ppm retention time error for MS and MS/MS with fragmentation pattern match were also considered as a criterion for identifying metabolites (Supplementary Table 1).

For data analysis, the list of identified metabolites in HILIC and C18 (both positive and negative mode) was combined, and duplicates were removed (Supplementary Table 2). MetaboAnalyst 5.0 was used for statistical and functional analysis like principal component analysis (PCA) and heat map analysis. Outlier intensities of metabolite(s) were excluded and the data were loaded in a matrix and statistical analysis was performed using peak intensities thereby using a statistical filter of interquartile range. Sample normalization was performed using “normalization by sum” methods followed by data transformation using log transformation (base 10) thereby scaling the data using Pareto scaling (mean-centered and divided by the square root of the standard deviation of each variable) (shown in MetaboAnalyst report, i.e., Supplementary Data sheet 3). Univariate analysis was performed using one-way analysis of variance (ANOVA) and post-hoc tests using a value of *p* (FDR) cutoff of ≤0.05. 2-D PCA was performed displaying 95% confidence regions to observe the inner clusters and find the apparent outliers. Hierarchical clustering heat maps were prepared using *t*-test/one-way ANOVA and Euclidean distance measure and Ward clustering method.

For quantitation, the peak intensity average of replicates for each metabolite extraction method (SC, FTC, and FTC + SC) was considered and used to identify differences among the extraction method. Chemical class-based analysis and functional categorization of metabolites were done using Metabolomics Workbench.^1^

### 2.5. Identification of AMR-associated metabolites

A majority of antibiotics work by targeting cellular processes like (1) cell wall synthesis, (2) cell metabolism, (3) nucleotide biosynthesis and/or (4) protein synthesis. So, it is evident that establishing and identifying metabolites associated with these processes can help us in understanding the metabolic fingerprint and mechanism of antibiotic resistance. Therefore, a literature search to identify the role of each metabolite in nucleotide synthesis, protein synthesis, cell wall biosynthesis, cell proliferation (potential representative of resistant phenotypes as they can grow even in presence of selective pressure, i.e., antibiotics) and cell death (representative drug sensitive bacterial population) was performed using Google Scholar and PubMed and the resulting information was utilized to fetch the AMR-associated metabolites and followed by data interpretation.

## 3. Results

### 3.1. Identification of metabolites

Metabolites extracted from *S. aureus* using three methods, FTC, SC, and their combination FTC + SC, were separated using UPLC ultimate 3,000 (C18and HILIC column), detected and quantified using an Orbitrap Fusion Mass Spectrometer and identified using the untargeted workflow of Progenesis QI software for metabolomics (Water Corporation). The list of identified non-polar and polar metabolites from the C18 and HILIC columns, respectively, was merged to get a collated list of metabolites extracted from each method-FTC, SC, and FTC + SC (Table 1).

**Table 1:** Number of identified metabolites with respect to different metabolite extraction methods and chromatographic surfaces (polar/non-polar).

Metabolite extraction method	Total metabolites identified from C18	Total metabolites identified from HILIC	Common among C18 and HILIC	Total metabolites
FTC	71	55	10	116
SC	68	61	10	119
FTC + SC	49	54	4	99
Total	188	171	24	335

The table contains all metabolites data, overlapping/similar metabolites were not removed while preparing this table.

**Figure 1:**
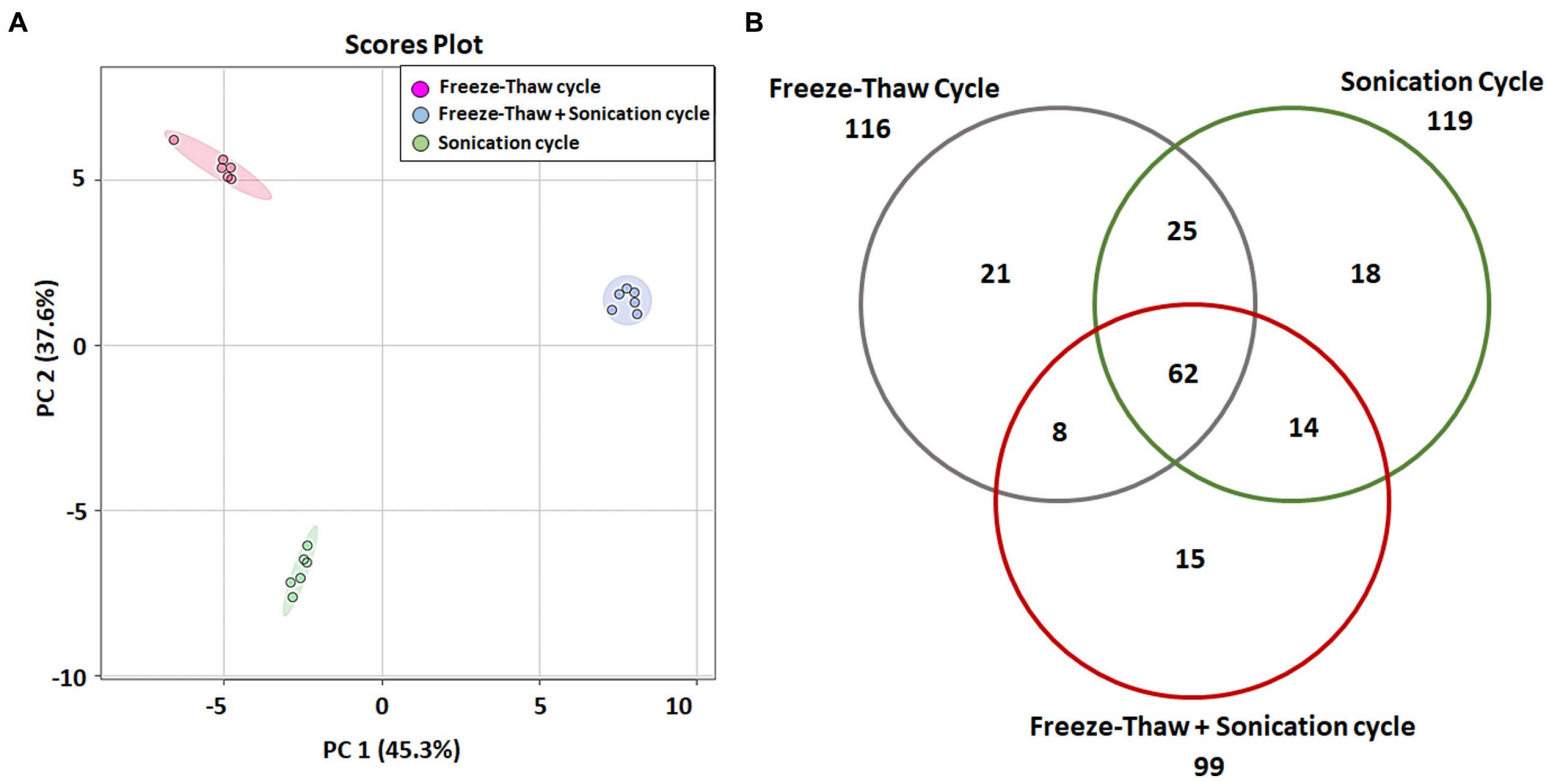
Multi-variate analysis of identified metabolites. **(A)** Principal component analysis (PCA) showing score plots of the metabolic differences between 3 methods (consisting a total 6 independent biological replicates for each method), red: freeze-thaw Cycle (FTC), green: Sonication Cycle (SC), blue: freeze-thaw Cycle followed by Sonication Cycle (FTC + SC). All three methods clustered distinctly having replicates of each method together indicating the consistency of sample preparation and downstream analysis. **(B)** Venn diagram representing unique and common metabolites among the three different methods of metabolite extraction [freeze-thaw cycle (FTC), Sonication Cycle (SC) and freeze-thaw cycle followed by Sonication Cycle (FTC + SC)]. A total of 62 metabolites were common among all 3 methods, whereas 21, 18, and 15 were uniquely identified in FTC, SC, and FTC + SC, respectively, indicating the potential biasness of the individual methods. Data from *n* ≥ 4 (out of six independent biological replicates were used in this analysis). One-way ANOVA was used to select the significant metabolites, i.e., 163 total metabolites (cutoff value of *p* ≤ 0.05) for the analysis.

The data of identified metabolites were subjected to PCA analysis to identify the distribution of the metabolomic profiles generated using FTC, SC and FTC + SC methods (Table 1). Noticeable differences between the three extraction protocols (3 distinct clusters) were observed indicating the characteristic fingerprint of each method (Figure 1A). The separation and clustering in PCA analysis also indicated the biasness of the individual method for different classes of metabolites and the quality of the sample generated for metabolomics experiments, respectively. Overall, a total of 116, 119, and 99 metabolites were identified using the FTC, SC, and FTC + SC methods of metabolite extraction, respectively, leading to the identification of 163 metabolites cumulatively (Tables 1, 2).

**Table 2:** List of identified metabolites specific to metabolite extraction protocols (FTC, SC, and FTC + SC) as well as commonly identified metabolites among all methods.

Common metabolites						Freeze thaw		Sonication		Freeze thaw + sonication	
S. no	Metabolite name	S. no	Metabolite name	S. no	Metabolite name	S. no	Metabolite name	S. no	Metabolite name	S. no	Metabolite name
1	1-(Carboxymethyl)cyclohexanecarboxylic Acid	22	Dipentyl phthalate	43	N-acetylmethionine	1	3-methyladenine	1	2-Hydroxy-4-methylpentanoic acid	1	16-Hydroxyhexadecanoic acid
2	1-Phenyl-2-butanone	23	Diphenylamine	44	N-acetylserine	2	3-Phosphoglyceric acid	2	Tetrahydroxy-5-alpha-pregnan-20-one 3,21-diacetate	2	1-Hydroxy-2-naphthoate
3	2′-Deoxyuridine 5′-mono-phos-phate	24	Elaidate	45	Nad	3	Abietic acid	3	3-Oxocholic acid	3	3-Hydroxy-3-methylglutaric acid
4	2-Methyl-S-benzothiazole	25	Ethyl paraben	46	N-alpha-acetyl-L-lysine	4	Alanine	4	4-{[3-(Diethylamino)propyl]amino}-4-oxobut-2-enoic acid	4	3-Hydroxypropanoic acid
5	2-Naphthalenesulfonic acid	26	Glucose	47	N-Epsilon-acetyllysine	5	Alloisoleucine	5	Acetylenedicarboxylic acid	5	Decanoate
6	3-Hydroxyphenylacetic acid	27	Glutamic acid	48	Norvaline	6	Azelate	6	Adipic acid	6	Erythronolactone
7	3-Tert-Butyladipic acid	28	Glutamine	49	Oleamide	7	Erucate	7	Dihydrouracil	7	Leucylproline
8	6-Carboxyhexanoate	29	Glyceraldehyde	50	O-phosphoserine	8	Glutamate	8	Ethylmethylacetic acid	8	Lithocholyltaurine
9	Ab-chminaca metabolite M6	30	Glyceric acid	51	Penbutolol	9	Leucine	9	Isoleucine	9	Melibiose
10	Adenine	31	Guanine	52	Phosphonoacetate	10	Mag	10	Linoleic acid	10	N-acetylaspartate
11	Adenosine monophosphate	32	Heptadecanoate	53	Phthalic acid	11	Methyl jasmonate	11	Melatonin	11	Peg N12
12	Alpha-lactose	33	Homoserine	54	Pyroglutamate	12	Nonanoate	12	N-acetyl-Dl-methionine	12	Suberic acid
13	Aspartate	34	Hydrochlorothiazide	55	Ribose	13	Palmitoleate	13	N-alpha-acetyl-L-asparagine	13	Succinate
14	Aspartic acid	35	Lactic acid	56	Salicylic acid	14	Peg N10	14	Peg N8	14	Triphenylphosphine oxide
15	Benzophenone	36	Malic acid	57	Serine	15	Pge2	15	P-toluenesulfonic acid		
16	Betaine	37	Myristate	58	Stearate	16	Prolylleucine	16	Risperidone		
17	Bmpea	38	N-(4-methoxy-5-morpholino-2-nitrophenyl)-N-(2-pyridyl)amine	59	Suberate	17	Stearic acid	17	Tartrate		
18	Carnitine	39	N-acetylaspartic acid	60	Uracil	18	Sucrose	18	Taurodeoxycholic acid		
19	Citroflex A-4	40	N-acetylhistidine	61	Uridine-5-monophosphate	19	Trehalose				
20	Dibutyl maleate	41	N-acetyl-L-aspartic acid	62	Urocanic acid	20	Tyrosine				
21	Dihydrosphingosine	42	N-acetyl-L-glutamine			21	Uridine 5′-diphospho-N-acetylglucosamine				

Overall, 62 metabolites were common among all three methods. FTC and SC yielded 25 common metabolites, 14 were common between FTC + SC and SC methods, and eight metabolites were identified by both FTC and FTC + SC methods (Figure 1B). Whereas 21 metabolites were uniquely identified in the FTC method followed by 18 unique metabolites by the sonication method, and only 15 unique metabolites by FTC + SC (Table 2).

### 3.2. Chemical classification of identified metabolites

**Figure 2:**
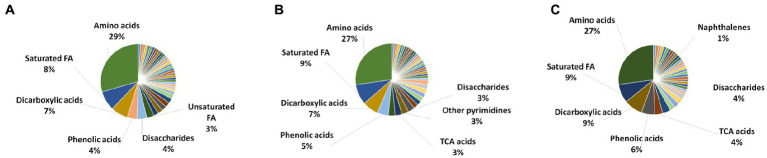
Chemical class analysis of identified metabolites. Pie-Chart of metabolite chemical class composition, identified using three different metabolite extraction methods *n* ≥ 4 (out of 6 independent experiments, selected using one-way ANOVA test with cutoff value of *p* ≤ 0.05) biological replicates were utilized to analyze the chemical class distribution. **(A)** freeze-thaw Cycle; **(B)** sonication Cycle; and **(C)** freeze-thaw Cycle followed by Sonication Cycle. The major metabolite class identified using each method was amino acids, followed by saturated fatty acids, dicarboxylic acids and phenolic acids (only major classes are shown in the figure). FA, fatty acids; TCA acids, trichloroacetic acids.

**Figure 3:**
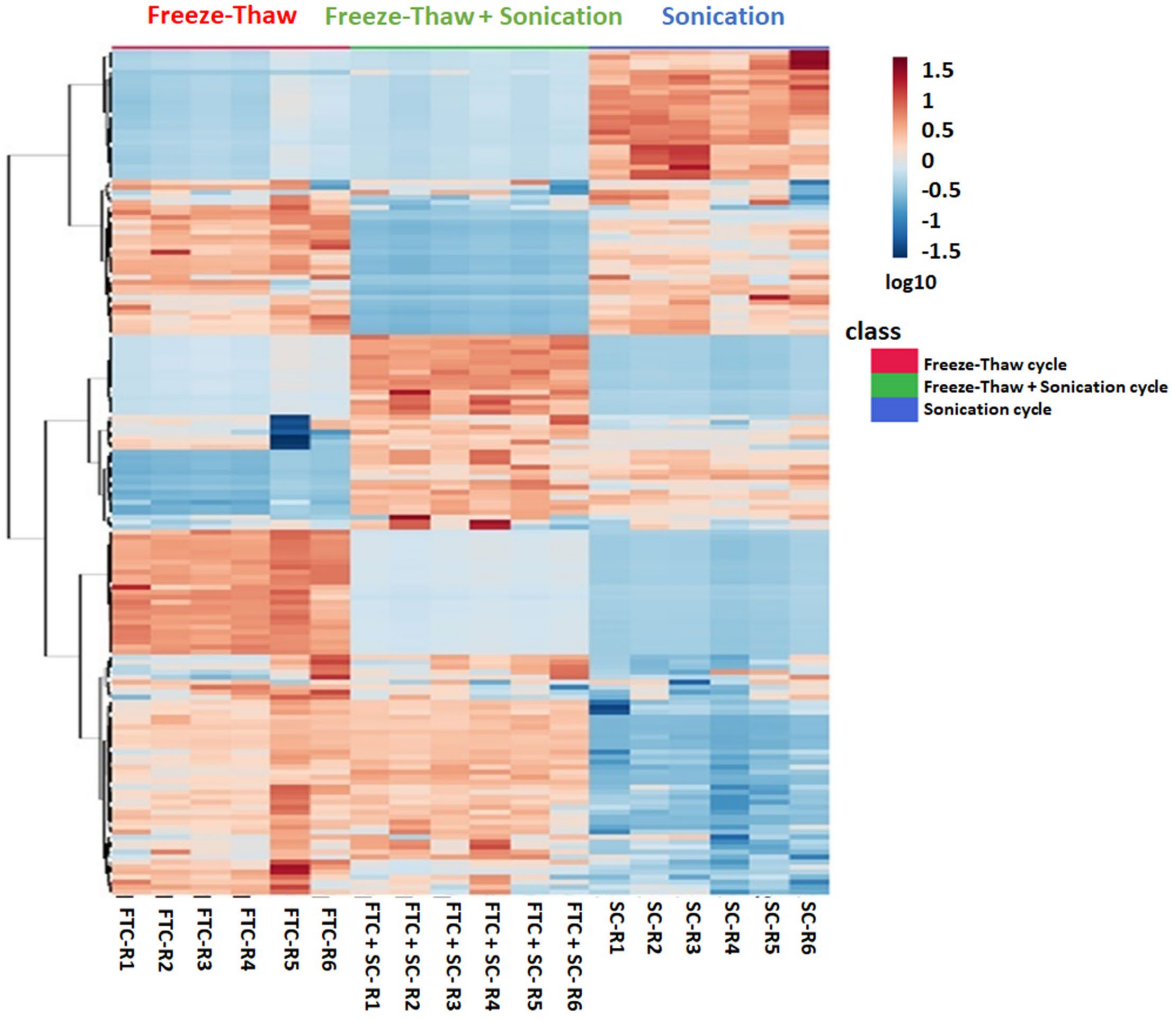
Heatmap profile depicting the relative expression levels of 163 metabolites (selected using one-way ANOVA test with cutoff value of *p* ≤ 0.05) from all three different extraction methods, i.e., freeze-thaw Cycle (FTC), Sonication Cycle (SC) and freeze-thaw Cycle followed by Sonication Cycle (FTC + SC) as indicated. Each column represents a specific biological replicates sample and row represent the metabolite. The raw intensities of metabolites from *n* ≥ 4 (from six independent biological replicates) were parsed by Pareto scaling (mean-centered and divided by the square root of the standard deviation of each variable) and rendered using the MetaboAnalyst 5.0 software. The clustering of the rows is based on Euclidean distance measure and Ward clustering method. A few metabolite clusters were observed to be more intensely expressed (indicated by Red) in a specific method compared to others showing biasness of the method(s). The information of the members in these clusters was utilized to explore their AMR-related biological function. The color is representing log10 transformed metabolite intensities (red: highest; blue: lowest).

**Figure 4:**
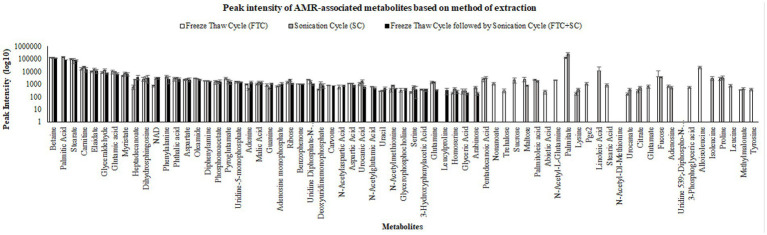
Pictorial representation of peak intensities of identified metabolites (selected using one-way ANOVA, means ± SD, with cutoff value of *p* ≤ 0.05) using three different metabolite extraction methods (FTC, SC and FTC + SC). The data represents the metabolites (intensity) from the *n* ≥ 4 (out of six independent biological replicate-Supplementary Table 1) samples. FTC, freeze-thaw cycle; SC, sonication cycle; FTC + SC, freeze-thaw cycle followed by sonication cycle.

All the metabolite extraction methods (FTC, SC, and FTC + SC) predominantly enabled the identification of different chemical classes of amino acids followed by saturated fatty acids and dicarboxylic acid [Figures 1–4 (Supplementary File-4)]. Similar pattern was observed in uniquely identified metabolites.

The dominant class of metabolites identified as amino acids (29% by FTC and 27% by SC and FTC + SC) followed by saturated fatty acids. Interestingly, the class “Naphthalene carboxylic acid” was uniquely identified in the FTC + SC method only (Figure 2). However, uniquely identified metabolites from individual methods showed biases toward specific chemical classes/subclass of metabolites (Figure 2).

### 3.3. Differential abundance of identified metabolites from FTC, SC, and FTC + SC method

The difference in intensity of metabolites was observed with different methods of extraction (Figure 3 and Supplementary Figure 5). A few metabolites were observed to be more intense in a specific method compared to others (Figure 4). For example, Alloisoleucine, nonanoate, palmitoleate, leucine, sucrose, tyrosine, glutamate, and prolylleucine were more abundant in the FTC method. Fucose, risperidone, hexazinone, and isoleucine were found to be more abundant in the SC method. Decanoate, succinate, leucylproline, and melibiose were found to be higher in intensity in FTC + SC. A few metabolites were identified by two methods. For example, maltose, suberate, pentadecanoic acid, palmitoleic acid, arachidate, fipronil, adenosine, and urocanate were more abundant in FTC and SC. Phenylalanine, pyruvic acid, sebacic acid, arabinose, palatinose, fumaric acid, laurate, palmitic acid, and serine were found to be more abundant in FTC + SC and SC methods. Guanine, azelaic acid, linoleate, adenine, and uracil were highly abundant in FTC and FTC + SC methods. Whereas a few metabolites like carnitine and glucose were identified by all three methods. This altogether indicates the biasness of metabolite extraction methods toward particular metabolite(s).

### 3.4. AMR-associated metabolites

Out of 163, a total of 69 metabolites were found to be related with AMR-associated phenotypes (cell proliferation and cell death) (Table 3). Of these, 57, 54, and 40 AMR-associated metabolites were found in metabolite samples extracted from FTC, SC or FTC + SC method, whereas 12, 7, and 2 were unique to each method (Table 3).

**Table 3:** Comparative overview of identified antimicrobial resistance (AMR)-associated metabolites among three different methods of metabolite extraction used in the study.

Peak intensity of AMR-associated metabolites and method of detection						
S. no.	Cell wall biosynthesis	Bacterial strain and species	FTC (Intensity ± standard deviation)	SC (Intensity ± standard deviation)	FTC + SC (Intensity ± standard deviation)	References
1	Pentadecanoic Acid	*Actinomycetes*	2,347 ± 849	3195.2 ± 602.5	-	Kumari et al. (2022)
2	Carnitine	*Escherichia coli*	16756.2 ± 3614.4	21936.2 ± 3016.3	14811.4 ± 5519.2	Eichler et al. (1996)
3	Phthalic acid	*Staphylococcus arlettae*	2709.1 ± 907.8	2901.3 ± 519	2382.4 ± 586	Acharyya et al. (2021)
4	Nonanoate	*Escherichia coli*	1076.4 ± 210.8	-	-	Lee et al. (2022)
5	Stearate	*Actinobacteria*	94998.9 ± 10206.5	77041.9 ± 35251.8	74,741 ± 10727.8	Tan et al. (2022)
6	Benzophenone	*Rhizobium*	1062.1 ± 51.5	1040.6 ± 48.1	1050.8 ± 69.4	Zhang et al. (2022)
7	Betaine	*Listeria monocytogenes*	125,016 ± 6628.1	124601.5 ± 11719.1	113966.6 ± 6,515	Whiteley et al. (2017)
8	Oleamide	*Pseudomonas aeruginosa*	2824.6 ± 98.3	2666.7 ± 212.2	2219.3 ± 210.5	Pyke (2022)
9	Trehalose	*Selaginella lepidophylla*	291.2 ± 97.4	-	-	Vanaporn and Titball (2020)
10	Sucrose	*Klebsiella pneumoniae*	2119.8 ± 923.8	-	-	Kumar et al. (2011)
11	Maltose	*Vibrio alginolyticus*	2505.4 ± 944.7	764.6 ± 113.8	-	Jiang et al. (2020)
12	Palmitoleic acid	*Mycobacterium tuberculosis*	2222.2 ± 239.6	1694.5 ± 307.5	-	Morris et al. (2005)
13	Uridine-5-monophosphate	*Pseudomonas aeruginosa*	1425.2 ± 212	1388.5 ± 239.1	1433.6 ± 81.9	Niu and Tan (2015)
14	Heptadecanoate	*Klebsiella pneumoniae*	618.1 ± 232.2	2260.7 ± 189.2	3408.2 ± 1251.1	Kumar et al. (2022)
15	Abietic Acid	*Streptococcus mutans*	248 ± 84.3	-	-	Ito et al. (2020)
16	Aspartic Acid	*Streptococcus faecalis ATCC 9790*	1116.5 ± 118.1	1126.7 ± 107	706.3 ± 125.8	Rahmanian et al. (1971)
17	Malic Acid	*Aeromonas hydrophila*	995.2 ± 147.4	1274.5 ± 363.4	1369.5 ± 276.3	Yao et al. (2016)
18	Dihydrosphingosine	*Porphyromonas gingivalis*	2520.4 ± 744.8	2611.6 ± 1170.9	3130.8 ± 1997	Ranjit et al. (2022)
19	Diphenylamine	*Bacillus licheniformis, Bacillus subtilis*	1731.9 ± 123.6	1826.2 ± 166.7	1594.6 ± 94	Salton and Schmitt (1967)
20	Elaidate	*Escherichia coli and Klebsiella pneumoniae*	9651.2 ± 1323.1	14,726 ± 3837.2	10965.1 ± 4004.4	Stahl et al. (2020)
21	Carvone	*Hafnia alvei*	779.6 ± 23.8	-	754.7 ± 30.6	Li et al. (2018)
22	Myristate	*Escherichia coli*	4434.1 ± 436.6	7039.8 ± 1414.1	6271.2 ± 1988.9	Somerville et al. (1996)
23	N-Acetylaspartic Acid	*Clostridium acetobutylicum*	618.1 ± 185.5	728.1 ± 80.1	719.4 ± 84.7	Reith et al. (2011)
24	N-Acetyl-L-Glutamine	*Escherichia coli*	2064.4 ± 122.4	-	-	Konopka (2012)
25	N-Acetylmethionine	*Escherichia coli*	345.2 ± 119.9	739.2 ± 55.4	451.4 ± 34.1	Viola et al. (2015)
26	Phosphonoacetate	*Pseudomonas fluorescens*	1423.2 ± 449	1464.8 ± 231	1560.7 ± 488.6	Kulakova et al. (2001)
27	Palmitic Acid	*Xanthomonas oryzae*	-	131,350 ± 12548.4	84982.3 ± 3847.9	Wang et al. (2021)
28	Palmitate	*Vibrio alginolyticus*	117837.2 ± 10,837	229951.2 ± 64518.4	-	Liu et al. (2019)
29	Aspartate	*Aeromonas hydrophila*	2142.3 ± 154.8	2472.7 ± 253.8	2237.4 ± 660.6	Zhao et al. (2018)
30	Lysine	*Thermotoga maritima, Escherichia coli*	172.9 ± 42.8	376.8 ± 110.7	-	Serganov et al. (2008)
31	Pge2	*Staphylococcus aureus*	1,066 ± 257.1	-	-	Wang et al. (2017)
32	Linoleic Acid	*Staphylococcus aureus*	-	11023.3 ± 11921.9	-	Antti et al. (2013)
33	Stearic Acid	*Vibrio* spp.	824.6 ± 213.6	-	-	Liu et al. (2019)
34	N-Acetyl-Dl-Methionine	*Escherichia coli*	-	530.2 ± 139.05	-	Usuda and Kurahashi (2005)
35	N-Acetylglutamic Acid	*Pseudomonas chlororaphis O6*	-	619.1 ± 53.9	483 ± 87.4	Park et al. (2018)
36	Uridine Diphosphate-N-Acetylglucosamine	*Corynebacterium glutamicum*	2239.6 ± 203.3	1523.1 ± 440.3	902.1 ± 143	Gauttam et al. (2021)
	Total		31	29	23	
Cellular metabolism						
37	Urocanate	*Pseudomonas aeruginosa*	168.8 ± 38.8	374.3 ± 95.7	-	Zhang et al. (2014)
38	Urocanic Acid	*Pseudomonas aeruginosa*	1092.1 ± 306.8	1677.2 ± 419.6	584.9 ± 119.5	Zhang et al. (2014)
39	Arabinose	*Mycobacterium tuberculosis*	-	520.8 ± 112.2	174 ± 51.3	Wolucka (2008)
40	Citrate	*Streptococcus diacetilactis*	280 ± 77.3	514.4 ± 179.7	-	Harvey and Collins (1963)
41	Glutamate	*Listeria monocytogenes*	646.1 ± 203.6	-	-	Feehily and Karatzas (2013)
42	Glutamine	*Salmonella*	1331.2 ± 303.9	1306.5 ± 162.6	336.7 ± 38.6	Yong et al. (2021)
43	Glyceric Acid	*Staphylococcus aureus and Pseudomonas aeruginosa*	277.9 ± 117.8	303.3 ± 92.7	175.8 ± 38.4	Thomas et al. (2016)
44	Leucylproline	*Bifidobacterium bifidum*	-	-	314 ± 175.9	Berg et al. (2015)
45	Fucose	*Klebsiella pneumoniae*	3968.6 ± 7013.9	3495.5 ± 511.1	-	Hudson (2022)
46	Glyceraldehyde	*Stenotrophomonas maltophilia*	8322.3 ± 1120.3	13281.7 ± 2500.2	6,880 ± 984.1	Gil-Gil et al. (2022)
	Total		6	8	6	
Nucleotide metabolism						
47	Adenosine	*Vibrio splendidus*	693 ± 154.7	530 ± 120.6	-	Li et al. (2023)
48	Ribose	*Staphylococcus aureus*	1413.4 ± 299.8	2034.2 ± 296.8	1068.7 ± 152.4	Baysarowich et al. (2008)
49	Adenine	*Escherichia coli*	933.4 ± 90.3	407 ± 70	1420.3 ± 346.2	Holt et al. (2017)
50	Adenosine monophosphate	*Salmonella enterica*	692 ± 81.8	786.2 ± 110.7	1086.6 ± 196	Pontes and Groisman (2019)
51	Guanine	*Staphylococcus aureus*	880.3 ± 227.9	487.4 ± 100.3	1099.8 ± 110.8	Dersch et al. (2017)
52	Uracil	*Methicillin-resistant Staphylococcus aureus (MRSA)*	281.8 ± 23.9	285.3 ± 36.5	476 ± 88.9	Fan et al. (2023)
53	Uridine 539;-Diphospho-N-Acetylglucosamine	*Bacillus subtilis*	2239.6 ± 203.3	-	-	Patel et al. (2023)
54	Deoxyuridine monophosphate	*Escherichia coli*	371.1 ± 54.3	1016.1 ± 482.1	772.9 ± 317.2	Zampieri et al. (2017)
	Total		7	7	5	
Protein synthesis						
55	3-Phosphoglyceric acid	*Aeromonas ca*via*e*	549.7 ± 96.2	-	-	Wang et al. (2023)
56	Glutamic acid	*Pseudomonas chlororaphis O6*	9577.1 ± 3,662	9769.6 ± 613.1	6497.5 ± 986.6	Park et al. (2018)
57	Alloisoleucine	*Clostridioides difficile*	20562.5 ± 4961.2	-	-	Robinson et al. (2019)
58	Isoleucine	*Edwardsiella piscicida*	-	2854.6 ± 951.3	-	Ye et al. (2018)
59	NAD	*Chromobacterium*	725 ± 147.2	2858.6 ± 494.9	2939.5 ± 412.2	Banerjee et al. (2017)
60	Proline	*Escherichia coli*	2674.8 ± 664.2	3196.7 ± 1046.7	-	Lin et al. (2019)
61	Leucine	*Edwardsiella piscicida*	737.4 ± 173.2	-	-	Ye et al. (2018)
62	3-Hydroxyphenylacetic Acid	*Pseudomonas aeruginosa*	360.5 ± 32.6	336.8 ± 54.9	346.3 ± 83.1	Pahalagedara et al. (2020)
63	Glycerophosphocholine	*Klebsiella pneumoniae and Mycoplasma*	339.6 ± 116.6	-	397 ± 77	Low et al. (2018)
64	Homoserine	*Variovorax paradoxus*	189.9 ± 39.3	402.8 ± 99.8	250.3 ± 78.4	Leadbetter and Greenberg (2000)
65	Methylmalonate	*Pseudomonas aeruginosa*	337.1 ± 29.1	436.1 ± 64.8	-	Su et al. (2010)
66	Pyroglutamate	*Saccharolobus solfataricus*	2748.9 ± 612.7	1738.5 ± 646.1	1,441 ± 537.4	Vetter et al. (2019)
67	Serine	*Edwardsiella piscicida*	215.4 ± 43.3	558.1 ± 178	352.5 ± 283.6	Ye et al. (2018)
68	Tyrosine	*Porphyromonas gingivalis*	367.2 ± 85	-	-	Whitmore and Lamont (2012)
69	Phenylalanine	*Escherichia coli and Pseudomonas putida*	-	3985.3 ± 1032.4	2567.6 ± 1449.6	Teufel et al. (2010)
	Total		13	10	8	

Shaded cells show the maximum intensity yielding method for each metabolite,—symbol shows metabolite not identified, (metabolite intensity data from *n* ≥ 4 out of six independent biological replicates) was considered while preparing this table. Supporting (raw data) data is available in (Supplementary Table 1).

## 4. Discussion

Infections caused by Gram-positive bacteria have become a serious public health threat causing high morbidity and mortality (Jubeh et al., 2020). Limited treatment options and rapid development of resistance to even the last-line antibiotics is the main reason for deaths, especially in the case of *S. aureus* (MRSA infections) (Chambers and Deleo, 2009; Alos, 2015). Besides enormous efforts, very few has been discovered over the last few decades, and this necessitates identifying new strategies to combat the emerging problem of AMR (WHO, 2020). Thus, understanding the molecular changes driving AMR among *S. aureus* becomes very important. Metabolomics can decode the real biochemical state of any organism and help in analyzing the emergence and/or spread of AMR phenomena (Pinu et al., 2019). To date metabolomics has been employed to identify new metabolic pathways (Deng et al., 2020), identification of bacterial species/strains (Zhang and Zhu, 2022), study the influence of external factors on bacteria (Tang, 2011), and in some cases reported to be of diagnostic use to detect bacterial infections (Fernández-García et al., 2018). However, so far, very few studies and efforts have been made to understand the spread and/or emergence of AMR and this may be primarily due to the unavailability of appropriate protocols and pipelines for investigating global and AMR-associated metabolites. As of now, even the performance of existing protocols for extracting AMR-associated intracellular metabolites from Gram-positive pathogen, *S. aureus*, are not established and hence the optimal method for the purpose remains unknown.

### 4.1. Analysis of metabolite extraction method and identified metabolites

**Figure 5:**
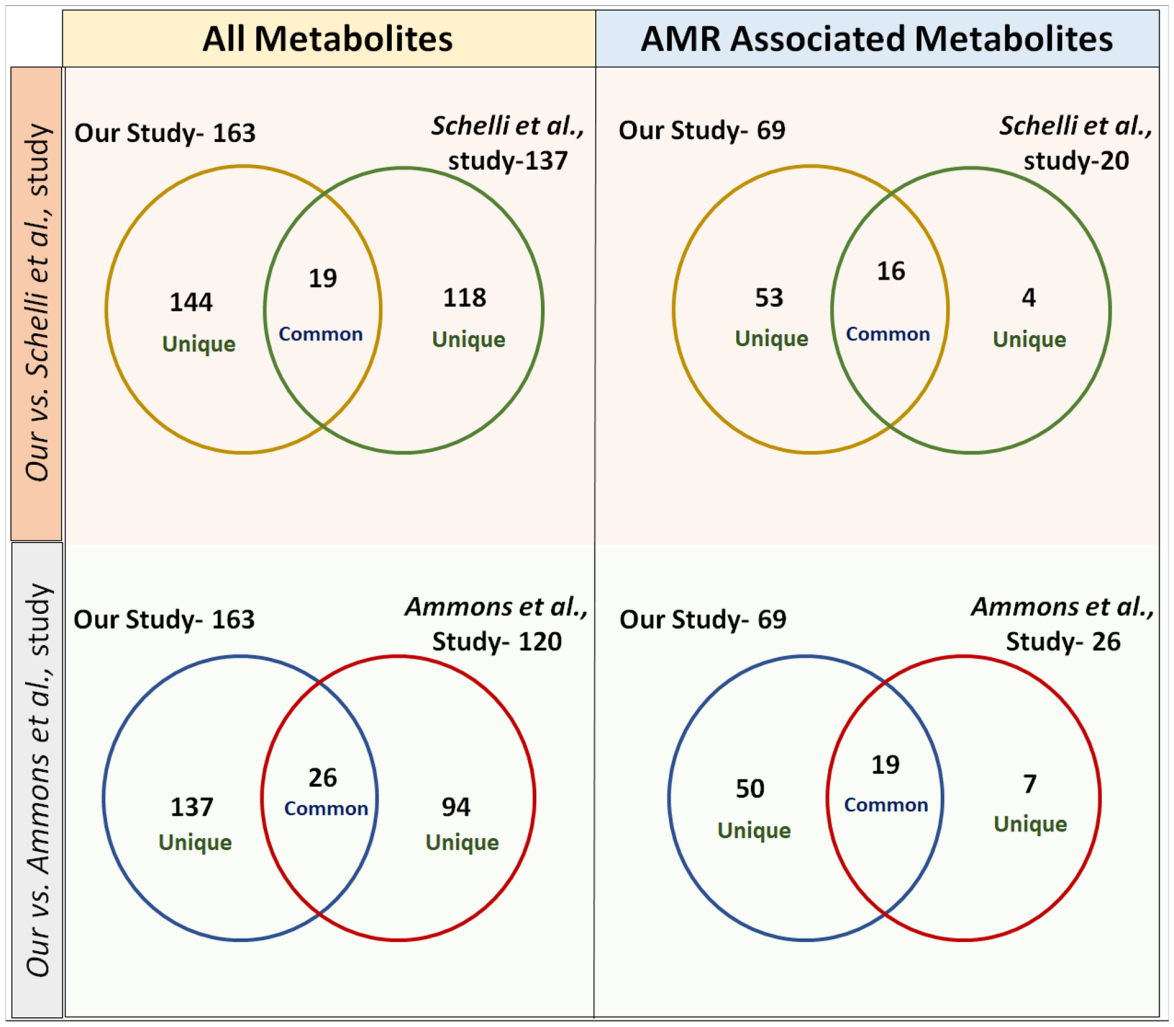
Venn diagrams representing a comparison of metabolites identified from our study versus previously published studies in the domain. In study-1 (Ammons et al.), 26 metabolites were found to be common in total metabolites and 19 metabolites were common in AMR-associated metabolites with our study. In study-2 (Schelli et al.), 19 metabolites were found to be common in total metabolites and 16 metabolites were common in AMR-associated metabolites with our study.

The metabolites were extracted from *S. aureus* (gentamicin and methicillin resistant) using the two most commonly used protocols, i.e., FTC, and SC, alone and in the combination [FTC followed by SC (FTC + SC)] and identified using ESI-LC–MS/MS (Orbitrap Fusion Tribrid Mass Analyzer), a highly sensitive and advanced mass-spectrometer to potentially achieve increased metabolomic coverage. Further, we have used 2 different Liquid Chromatography (LC) columns, i.e., HILIC and C18 which are specific for separating compounds with different physico-chemical properties were also used. Generally, HILIC columns are very well known for separating polar amino acids, organic acids, sugars, phosphorylated sugars, nucleobases, nucleotides, phosphorylated metabolites, hydrophilic vitamins, and coenzymes (Galeano Garcia et al., 2019). C18 columns are usually employed to separate semi-polar and non-polar compounds like alkaloids, flavonoids, phenolic acids, and other glycosylated species (Liu et al., 2019). Data analysis from individual LC columns (HILIC and C18) revealed that C18 enabled a higher number of metabolite identification compared to HILIC among all the metabolite extraction methods used (Table 1). This may potentially be because of poor retention of very polar metabolites during chromatographic separation and hence is in line with published literature (Harrieder et al., 2022). Only a few metabolites were common among the metabolites identified using C18 and HILIC columns. A total of 163 metabolites were cumulatively identified using all chromatographic surfaces and extraction methods. The SC method showed the highest number of metabolites though comparable with FTC among the total identified metabolites (116 by FTC and 119 by SC out of a total of 163 metabolites). The most likely reason for low yield in FTC + SC methods might be the degradation of already extracted metabolites during sonication. Among 163 metabolites, 21, 18, and 15 were uniquely identified in FTC, SC, and FTC + SC methods indicating the potential bias of the method toward specific metabolite(s) (Table 2). Chemical class-based functional categorization and analysis revealed that amino acids were the major chemical class of metabolites identified by all methods. The FTC method was observed to be more biased toward saturated fatty acids (17%), and disaccharides (17%) (Supplementary Figure 2). The SC method showed biasness toward hexoses (2%), C24 bile acids (2%), and pyrimidines (3%) (Supplementary Figure 3). Disruption of the peptidoglycan layer (mesh-like network of amino acids and sugar) during metabolite extraction might be the possible reason for yielding a high number of amino acids, dipeptides, sugar derivatives like hexoses, disaccharides, and sugar acids (Vollmer et al., 2008). However, a combination of FTC + SC methods showed an enrichment of hydroxy fatty acids (28%) (Supplementary Figure 4). This altogether indicates the potential bias of each method for investigating specific metabolites or classes of metabolites. Therefore, the method of metabolite extraction shall primarily be chosen based on the specific metabolite(s) of interest or classes of metabolites. A few attempts have been made by researchers to establish the metabolome of this clinically relevant Gram-positive pathogen, *S. aureus*. Recently, 109 metabolites (RN450) and 107 (450 M) metabolites were identified using cold methanol and vortexing (vigorously for ~1 min) based metabolite extraction method with HPLC coupled with a TSQ Quantiva Triple Quadrupole mass spectrometer in (methicillin-resistant *S. aureus*) MRSA and (methicillin sensitive *S. aureus*) MSSA strains, respectively, after exposure to a sublethal dose of antibiotic (ampicillin, kanamycin, norfloxacin) (Schelli et al., 2017). However, the number of identified metabolites was low compared to our study potentially due to the use of only the HILIC column, one extraction method, i.e., vortexing (instead of freeze-thaw and vortexing or sonication) and the low-sensitive equipment (Schelli et al., 2017). Another attempt to differentiate MSSA and MRSA biofilm and planktonic phenotypes using an (Nuclear magnetic resonance spectroscopy) NMR-based metabolomics study has been reported. They have identified a total of 120 metabolites (Ammons et al., 2014). Although the reason for identifying a lower number of metabolites in this study might be because of the technology used, NMR is already known to be less sensitive than ESI-LC–MS/MS (Ammons et al., 2014). Among them, 19 and 26 common metabolites were identified when compared to our study, respectively, (Table 4 and Figure 5) (Ammons et al., 2014; Schelli et al., 2017). Similarly, a total of 173 metabolites were identified in MRSA and MSSA using a combination of hydrophilic interaction liquid chromatography and PFP columns (pentafluorophenyl-propyl) coupled with high-resolution mass spectrometry (Aros-Calt et al., 2019). The possible reason for yielding a higher number of metabolites in comparison to our study may be due to a bigger library size compared to our in-house library (~950 metabolites). Notably, none of the above-discussed studies have mentioned the identified AMR-associated metabolites.

**Table 4:** Comparison of metabolites [total and antimicrobial resistance (AMR) associated] identified from our study versus other reported studies.

All metabolites				
Total metabolites identified in Schelli et al. study Schelli et al. (2017)	Common metabolites identified in Schelli et al. versus our study	Total metabolites identified in our study	Common metabolites identified in Ammons et al. versus our study	Total metabolites identified in Ammons et al. study Ammons et al. (2014)
137	19	163	26	120
AMR associated metabolites				
20	16	69	19	26

### 4.2. Analysis of AMR-associated metabolites

Of the identified 163 metabolites, 69 (42.0%) were observed to be associated with AMR in published literature (Table 3). Among these, the majority of identified AMR-associated metabolites were primarily linked with cell wall biosynthesis (52.1%), followed by cellular metabolism (14.4%), nucleotide biosynthesis (11.5%), and protein synthesis (21.7%) indicating the potential bias of the method of metabolite extraction or the technique. A comparison of identified AMR-associated metabolites list with previously reported study has shown only 16 and 19 common metabolites (Table 4) (Ammons et al., 2014; Schelli et al., 2017). Interestingly, the FTC method yielded a slightly higher number of AMR-associated metabolites (57 metabolites) compared to SC (54 metabolites) with 42 commonly identified metabolites. Whereas only 40 AMR-associated metabolites were identified using the FTC + SC method potentially due to the degradation of extracted metabolites (after FTC) during sonication. This altogether indicates the suitability of both the methods, FTC or SC, for investigating AMR-associated metabolites in *S. aureus* and Gram-positive pathogens; however, the metabolite of interest shall be key in choosing the method for metabolite extraction.

### 4.3. Analysis of metabolite extraction methods between Gram-positive pathogen, *Staphylococcus aureus* and Gram-negative pathogen, *Klebsiella pneumoniae*

In this investigation, the maximum number of metabolites from *S. aureus* were identified using SC (119 metabolites) and subsequently by FTC (116 metabolites) and FTC + SC (99 metabolites). Whereas for *K. pneumoniae*, the maximum number of metabolites were identified by the FTC (151) method followed by FTC + SC (132 metabolites) and SC (103 metabolites; Supplementary Table 2) (Kumar et al., 2022). Notably, the number of metabolites identified in *K. pneumoniae* was more when compared to *S. aureus* and the potential reason remains unknown. Among these, only a few metabolites were commonly identified between *S. aureus* and *K. pneumoniae* (26 by FTC, 45 by SC and 31 by FTC + SC). A similar pattern was observed in AMR-associated metabolites. This supports that the metabolomic architecture and AMR-associated metabolites among *S. aureus* (a Gram-positive pathogen) may be different than the *K. pneumoniae* (a Gram-negative pathogen). This also suggests that the optimal method for extracting metabolites from Gram-positive pathogens may be different than the method for Gram-negative pathogens.

## 5. Conclusion

The increasing emergence of AMR among Gram-positive pathogens such as *S. aureus* is becoming a global health concern and urgently demands strategies to control it. Understanding the metabolomic footprint of antibiotic-resistant/sensitive pathogens has been shown to improve our understanding of the emergence/spread of AMR superbugs. However, very few efforts have been made in this direction possible due to the lack of appropriate methods of metabolite extraction.

Therefore, in this investigation, we have compared the performance of the two most common methods, i.e., FTC and SC alone and in combination (FTC + SC), for extracting metabolites from *S. aureus* (gentamicin and methicillin-resistant) using a highly sensitive and advanced HPLC-coupled mass-spectrometer (ESI-LC–MS/MS). The SC and FTC methods were observed to identify a comparable number of total metabolites as well as AMR-associated metabolites and hence may be utilized for investigating the metabolome of *S. aureus* or other Gram-positive bacteria after further validation. FTC + SC gave a lower yield of metabolites possible due to the degradation of already extracted metabolites by FTC during sonication. The methods of metabolite extraction were also observed to have biasness toward specific metabolite(s) or class of metabolites (“Tryptamines” was unique to the SC method, “C20 isoprenoids and Jasmonic acids” were unique to FTC, and “Naphthalenes” were unique to FTC + SC method) and hence can potentially impact the overall finding of the metabolomics-based studies. Therefore, the method of metabolite extraction shall be primarily chosen based on the metabolites of interest in the investigation. Altogether, our data can help in designing/planning pathway specific/directed metabolomics studies which could improve understanding of the emergence/spread of AMR superbugs and ultimately contribute to improving the efficacy of existing antimicrobial therapies.

## Data availability statement

The datasets presented in this study can be found in online repositories (MetaboLights study identifier-MTBLS7338). The names of the repository/repositories and accession number(s) can be found in the article/Supplementary material.

## Author contributions

NK conceived, designed, and coordinated the study. SK and MK generated the study material. AK and YK performed most of the experiments. RS, LT, AK SK, YK, and NK performed the data analysis. LT, RS, and SR wrote the manuscript. NK and YK supervised the manuscript writing and revisions. All authors contributed to the article and approved the submitted version.

## Funding

This work was supported by grants awarded to NK from the Indian Council of Medical Research Govt. of India (Grant No. AMR/Adhoc/233/2020-ECD-II) and Translational Health Science and Technology Institute Faridabad, India. RS received Senior Research Fellowship from the Council of Scientific and Industrial Research, Govt. of India, and SS and LT received Junior Research Fellowship from the Department of Biotechnology, Govt. of India.

## Conflict of interest

The authors declare that the research was conducted in the absence of any commercial or financial relationships that could be construed as a potential conflict of interest.

## Publisher’s note

All claims expressed in this article are solely those of the authors and do not necessarily represent those of their affiliated organizations, or those of the publisher, the editors and the reviewers. Any product that may be evaluated in this article, or claim that may be made by its manufacturer, is not guaranteed or endorsed by the publisher.

## Footnotes

^1^ https://www.metabolomicsworkbench.org/
