# Supplementary material for: Comparison of freeze-thaw and sonication cycle-based methods for extracting AMR-associated metabolites from Staphylococcus aureus
Source: Front Microbiol. 2023 Apr 27;14:1152162. doi: 10.3389/fmicb.2023.1152162 (PMC10174324; doi:10.3389/fmicb.2023.1152162)
Supplement: Supplementary file 3 [file Data_Sheet_1.PDF]

Metabolomic Data Analysis with MetaboAnalyst 5.0

Name: guest7902052904536063477

March 23, 2023

1 Data Processing and Normalization

1.1 Reading and Processing the Raw Data

MetaboAnalyst accepts a variety of data types generated in metabolomic studies, including compound concentration data, binned NMR/MS spectra data, NMR/MS peak list data, as well as MS spectra (NetCDF, mzXML, mzDATA). Users need to specify the data types when uploading their data in order for MetaboAnalyst to select the correct algorithm to process them. Table 1 summarizes the result of the data processing steps.

1.1.1 Reading Peak Intensity Table

The peak intensity table should be uploaded in comma separated values (.csv) format. Samples can be in rows or columns, with class labels immediately following the sample IDs.

Samples are in columns and features in rows. The uploaded file is in comma separated values (.csv) format. The uploaded data file contains 18 (samples) by 163 (peaks(mz/rt)) data matrix.

1.1.2 Data Integrity Check

Before data analysis, a data integrity check is performed to make sure that all the necessary information has been collected. The class labels must be present and contain only two classes. If samples are paired, the class label must be from -n/2 to -1 for one group, and 1 to n/2 for the other group (n is the sample number and must be an even number). Class labels with same absolute value are assumed to be pairs. Compound concentration or peak intensity values should all be non-negative numbers. By default, all missing values, zeros and negative values will be replaced by the half of the minimum positive value found within the data (see next section)

1.1.3 Missing value imputations

Too many zeroes or missing values will cause difficulties for downstream analysis. MetaboAnalyst offers several different methods for this purpose. The default method replaces all the missing and zero values with a small values (the half of the minimum positive values in the original data) assuming to be the detection limit. The assumption of this approach is that most missing values are caused by low abundance metabolites (i.e.below the detection limit). In addition, since zero values may cause problem for data normalization (i.e. log), they are also replaced with this small value. User can also specify other methods, such as replace by mean/median, or use K-Nearest Neighbours (KNN), Probabilistic PCA (PPCA), Bayesian PCA (BPCA) method, Singular Value Decomposition (SVD) method to impute the missing values [1]. Please choose the one that is the most appropriate for your data.

[1] Stacklies W, Redestig H, Scholz M, Walther D, Selbig J. *pcaMethods: a bioconductor package, providing PCA methods for incomplete data.*, Bioinformatics 2007 23(9):1164-1167

Zero or missing values were replaced by 1/5 of the min positive value for each variable.

1.1.4 Data Filtering

The purpose of the data filtering is to identify and remove variables that are unlikely to be of use when modeling the data. No phenotype information are used in the filtering process, so the result can be used with any downstream analysis. This step can usually improves the results. Data filter is strongly recommended for datasets with large number of variables (> 250) datasets contain much noise (i.e.chemometrics data). Filtering can usually improve your results[2].

*For data with number of variables < 250, this step will reduce 5% of variables; For variable number between 250 and 500, 10% of variables will be removed; For variable number bwteen 500 and 1000, 25% of variables will be removed; And 40% of variabled will be removed for data with over 1000 variables. The None option is only for less than 5000 features. Over that, if you choose None, the IQR filter will still be applied. In addition, the maximum allowed number of variables is **10000***

Feature filtering based on Interquantile Range

Table 1: Summary of data processing results

	Features (positive)	Missing/Zero	Features (processed)
FTC_R1	116	47	163
FTC_R2	116	47	163
FTC_R3	116	47	163
FTC_R4	116	47	163
FTC_R5	112	51	163
FTC_R6	115	48	163
FTC+SC_R1	99	64	163
FTC+SC_R2	99	64	163
FTC+SC_R3	99	64	163
FTC+SC_R4	99	64	163
FTC+SC_R5	99	64	163
FTC+SC_R6	99	64	163
SC_R1	120	43	163
SC_R2	120	43	163
SC_R3	119	44	163
SC_R4	120	43	163
SC_R5	120	43	163
SC_R6	120	43	163

[2] Hackstadt AJ, Hess AM.*Filtering for increased power for microarray data analysis*, BMC Bioinformatics. 2009; 10: 11.

1.2 Data Normalization

The data is stored as a table with one sample per row and one variable (bin/peak/metabolite) per column. The normalization procedures implemented below are grouped into four categories. Sample specific normalization allows users to manually adjust concentrations based on biological inputs (i.e. volume, mass); row-wise normalization allows general-purpose adjustment for differences among samples; data transformation and scaling are two different approaches to make features more comparable. You can use one or combine both to achieve better results.

The normalization consists of the following options:

1. Row-wise procedures:
    - Sample specific normalization (i.e. normalize by dry weight, volume)
    - Normalization by the sum
    - Normalization by the sample median
    - Normalization by a reference sample (probabilistic quotient normalization)[3]
    - Normalization by a pooled or average sample from a particular group
    - Normalization by a reference feature (i.e. creatinine, internal control)
    - Quantile normalization
2. Data transformation :
    - Log transformation (base 10)
    - Square root transformation
    - Cube root transformation
3. Data scaling:
    - Mean centering (mean-centered only)
    - Auto scaling (mean-centered and divided by standard deviation of each variable)
    - Pareto scaling (mean-centered and divided by the square root of standard deviation of each variable)
    - Range scaling (mean-centered and divided by the value range of each variable)

Figure 1 shows the effects before and after normalization.

[3]Dieterle F, Ross A, Schlotterbeck G, Senn H. *Probabilistic quotient normalization as robust method to account for dilution of complex biological mixtures. Application in 1H NMR metabonomics*, 2006, Anal Chem 78 (13);4281 - 4290

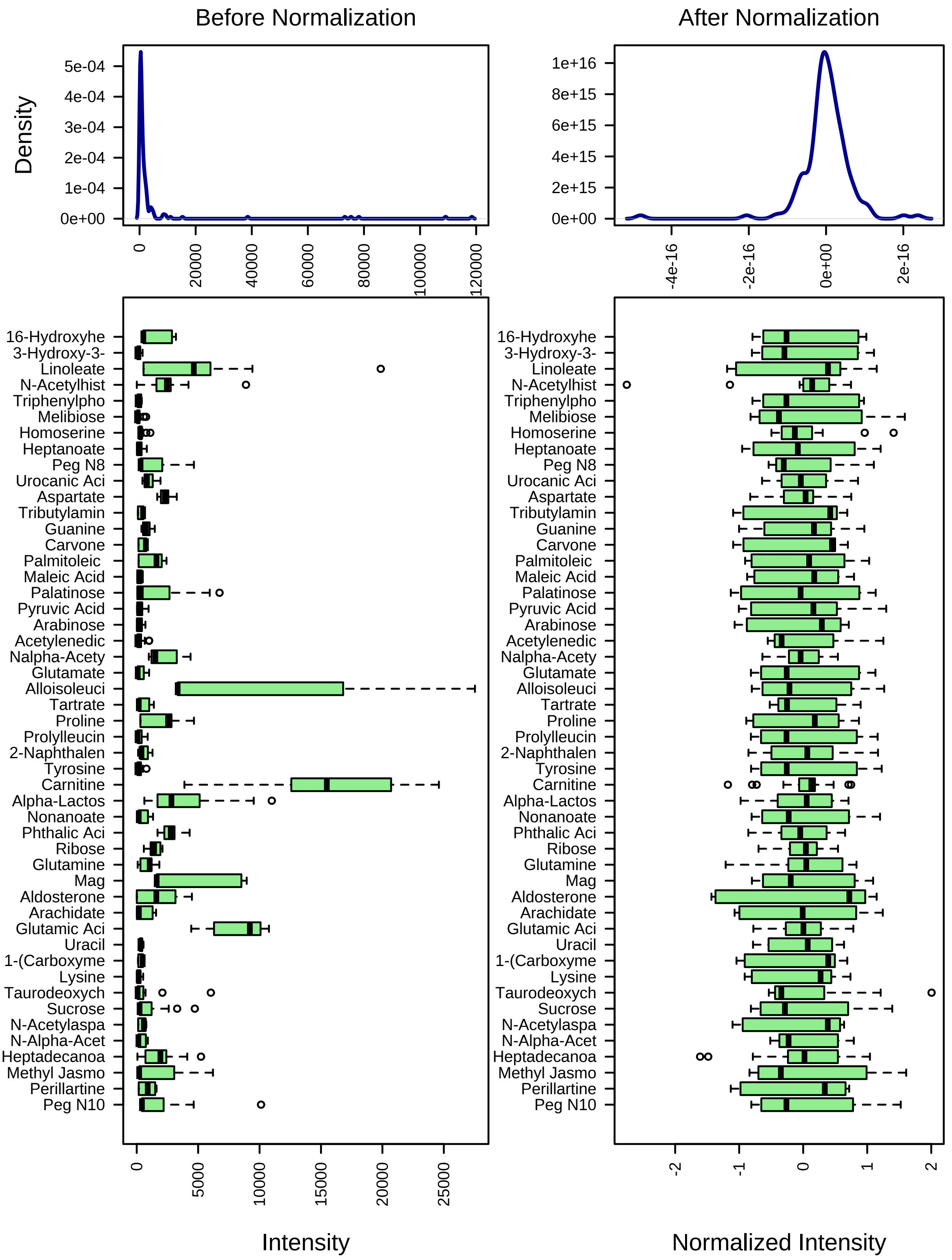

Figure 1: Box plots and kernel density plots before and after normalization. The boxplots show at most 50 features due to space limit. The density plots are based on all samples. Selected methods : Row-wise normalization: Normalization to constant sum; Data transformation: Log10 Normalization; Data scaling: Pareto Scaling.

2 Statistical and Machine Learning Data Analysis

MetaboAnalyst offers a variety of methods commonly used in metabolomic data analyses. They include:

1. Univariate analysis methods:
   - Fold Change Analysis
   - T-tests
   - Volcano Plot
   - One-way ANOVA and post-hoc analysis
   - Correlation analysis
2. Multivariate analysis methods:
   - Principal Component Analysis (PCA)
   - Partial Least Squares - Discriminant Analysis (PLS-DA)
3. Robust Feature Selection Methods in microarray studies
   - Significance Analysis of Microarray (SAM)
   - Empirical Bayesian Analysis of Microarray (EBAM)
4. Clustering Analysis
   - Hierarchical Clustering
     - Dendrogram
     - Heatmap
   - Partitional Clustering
     - K-means Clustering
     - Self-Organizing Map (SOM)
5. Supervised Classification and Feature Selection methods
   - Random Forest
   - Support Vector Machine (SVM)

`Please note:  some advanced methods are available only for two-group sample analyais.`

3 Appendix: R Command History

```
 [1] "mSet<-InitDataObjects(\"pktable\", \"stat\", FALSE)"
 [2] "mSet<-Read.TextData(mSet, \"Replacing_with_your_file_path\", \"colu\", \"disc\");"
 [3] "mSet<-SanityCheckData(mSet)"
 [4] "mSet<-ReplaceMin(mSet);"
 [5] "mSet<-SanityCheckData(mSet)"
 [6] "mSet<-FilterVariable(mSet, \"iqr\", 5, \"F\", 25, F)"
 [7] "mSet<-PreparePrenormData(mSet)"
 [8] "mSet<-Normalization(mSet, \"SumNorm\", \"LogNorm\", \"ParetoNorm\", ratio=FALSE, ratioNum=20)"
 [9] "mSet<-PlotNormSummary(mSet, \"norm_0_\", \"png\", 72, width=NA)"
[10] "mSet<-PlotSampleNormSummary(mSet, \"snorm_0_\", \"png\", 72, width=NA)"
[11] "mSet<-SaveTransformedData(mSet)"
[12] "mSet<-PreparePDFReport(mSet, \"guest7902052904536063477\")\n"
```

The report was generated on Thu Mar 23 02:22:48 2023 with R version 4.2.2 (2022-10-31), OS system: Linux, version: -Ubuntu SMP Wed Feb 22 14:14:39 UTC 2023 .
