# Supplementary material for: Comparison of freeze-thaw and sonication cycle-based methods for extracting AMR-associated metabolites from Staphylococcus aureus
Source: Front Microbiol. 2023 Apr 27;14:1152162. doi: 10.3389/fmicb.2023.1152162 (PMC10174324; doi:10.3389/fmicb.2023.1152162)
Supplement: Supplementary file 4 [file Data_Sheet_2.docx]

**Supplementary File-1**

**
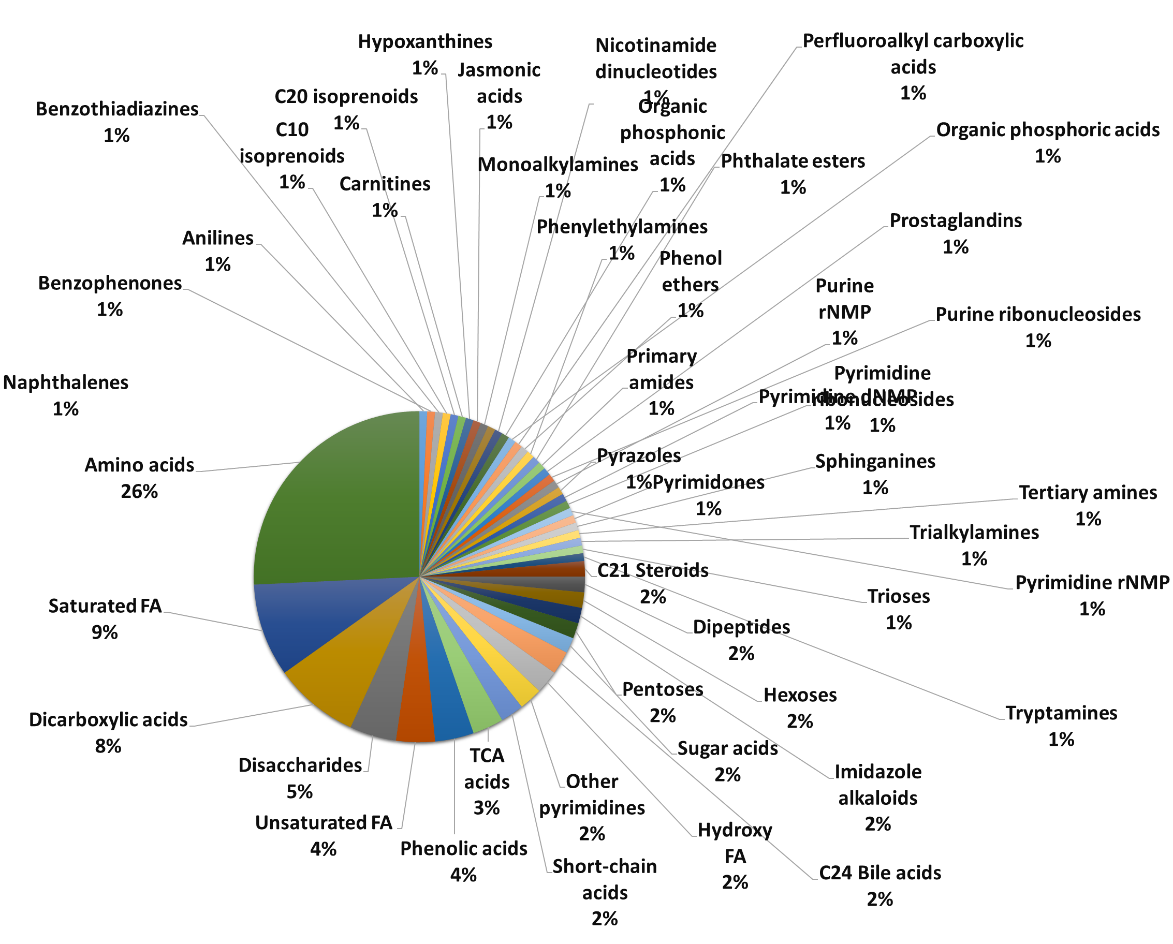
**

**Supplementary Figure-1:** Chemical class analysis of identified metabolites for all metabolites (All methods, FTC, SC, FTC+SC, -total 163 metabolites). One-way ANOVA with a cutoff p-value of ≤0.05 was used (from n ≥ 4 out of 6 independent experiments) in this analysis. FTC: Freeze-Thaw Cycle, SC: Sonication Cycle, FTC+SC: Freeze-Thaw Cycle followed by Sonication Cycle.

**
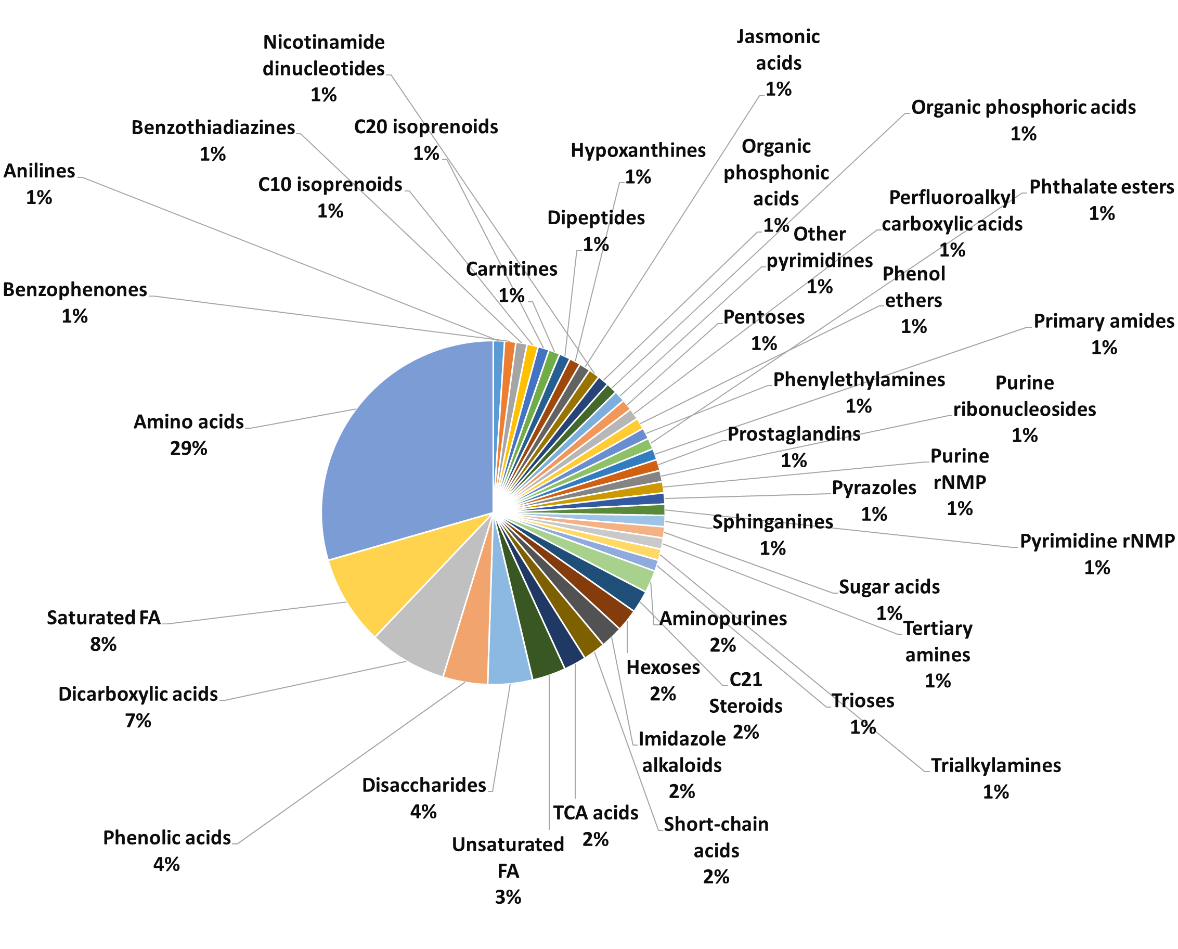
**

**Supplementary Figure-2:** Chemical class analysis of identified metabolites using Freeze-Thaw cycle (116 from a total of 163 metabolite). One-way ANOVA with a cutoff p-value of ≤0.05 was used (from n ≥ 4 out of 6 independent experiments) in this analysis.


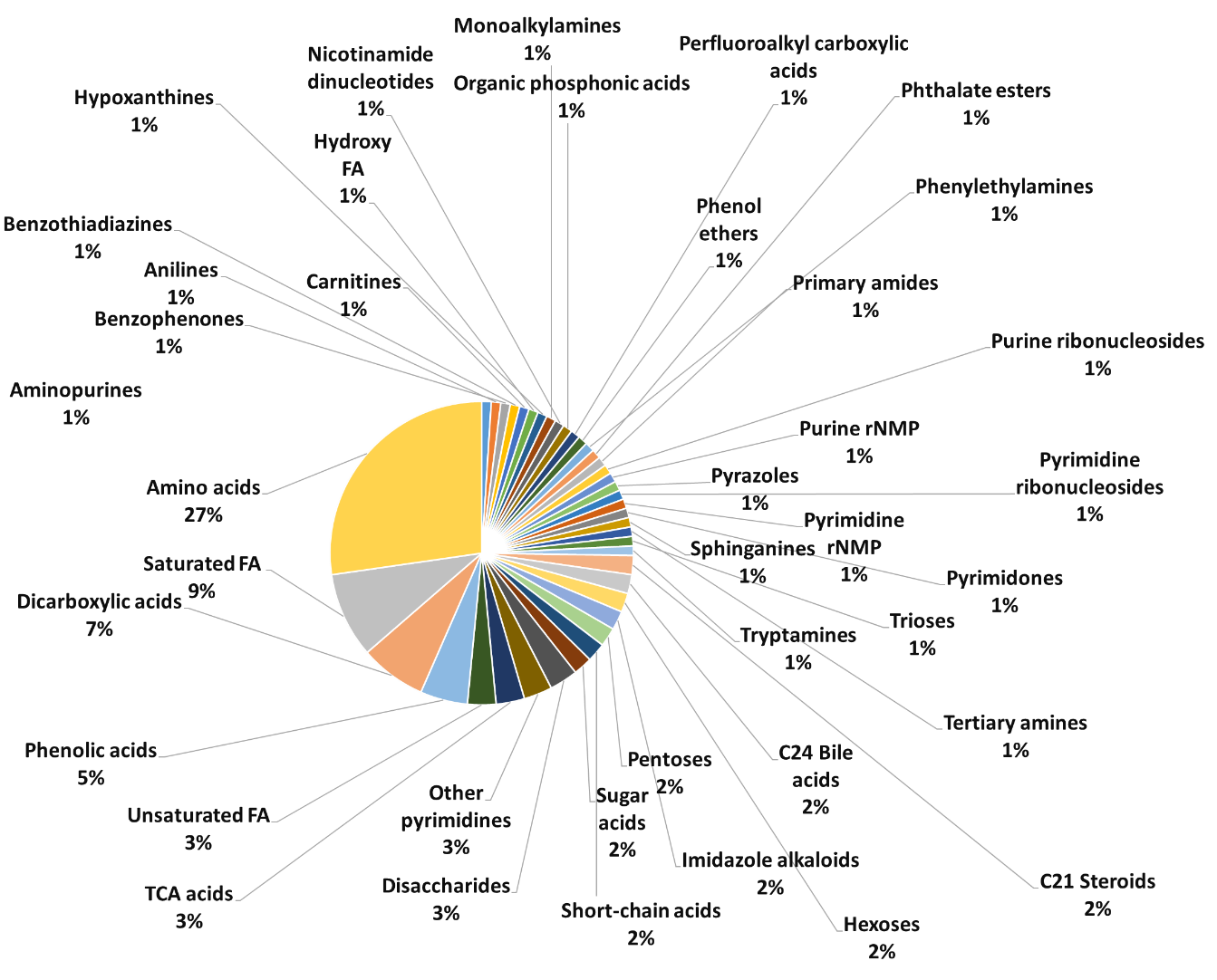


**Supplementary Figure-3:** Chemical class analysis of identified metabolites using Sonication Cycle (119 from 163 total metabolites). One-way ANOVA with a cutoff p-value of ≤0.05 was used (from n ≥ 4 out of 6 independent experiments) in this analysis.


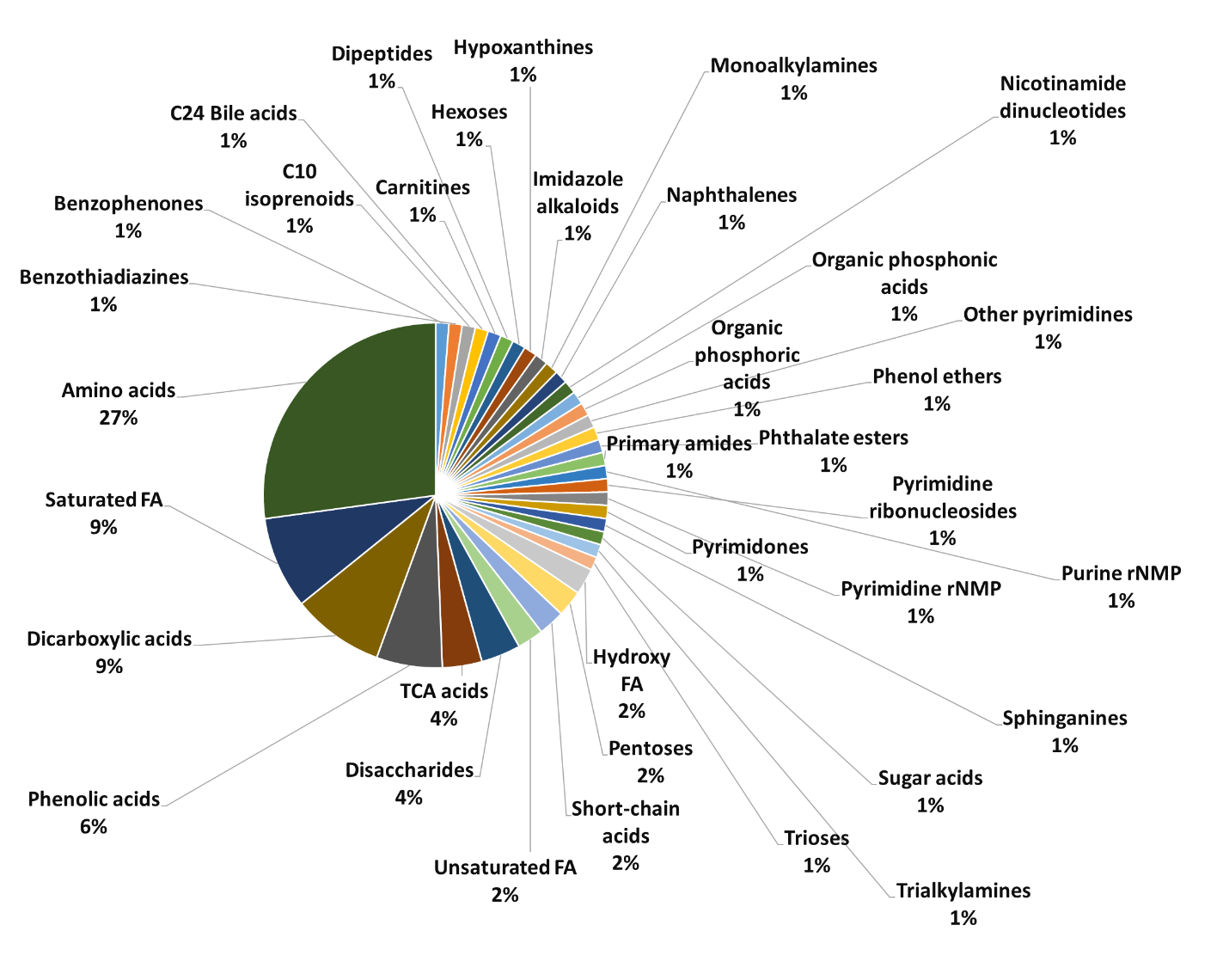


**Supplementary Figure-4:** Chemical class analysis of identified metabolites using Freeze-Thaw Cycle followed by sonication cycle (99 from 163 total metabolites). One-way ANOVA with a cutoff p-value of ≤0.05 was used (from n ≥ 4 out of 6 independent experiments) in this analysis.


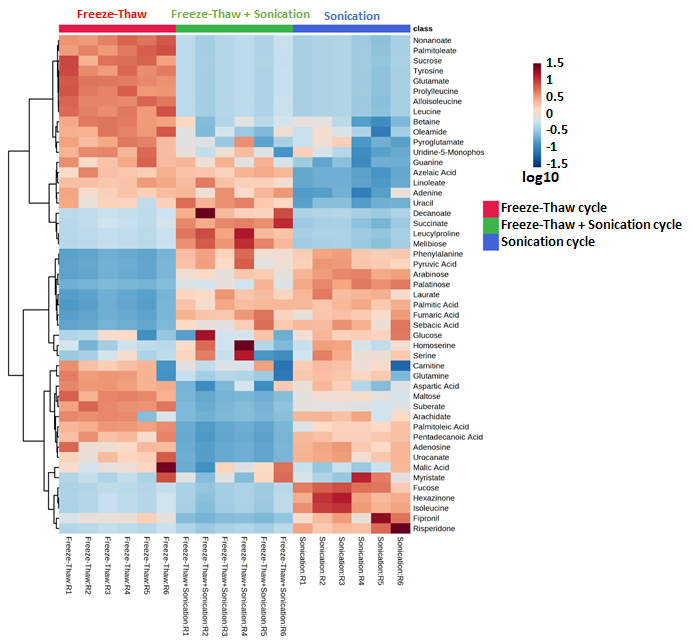


**Supplementary Figure-5:** Heatmap representation of differential abundance of metabolites (49 metabolites from a total of 163 metabolites (selected using one-way ANOVA test with cutoff p-value ≤0.05 and n≥4 out of 6 independent biological replicate) that are discussed in the manuscript due to their differential expression among different methods of metabolite extraction (Freeze-Thaw (FTC), Sonication (SC) and Freeze-Thaw + Sonication (FTC+SC)) and/or their relevance with AMR-related research. Each column represent a specific biological replicates sample and row represent the metabolite. The color represents the log10 transformed metabolite intensity (red: highest; blue: lowest).
